# Exploring the Neuroprotective Effects of Xanthohumol and Quercetin for the treatment of Alzheimer's Disease

**DOI:** 10.1002/alz70857_101587

**Published:** 2025-12-25

**Authors:** Bushra Bashir, Sachin Kumar Singh, Monica Gulati, Sukriti Vishwas, Nikhil B Khandale, Biswajit Pal, Sambita Panda

**Affiliations:** ^1^ School of Pharmaceutical Sciences, Lovely Professional University, Jalandhar, Punjab, India; ^2^ Lovely Professional University, Phagwara, Punjab, India

## Abstract

**Background:**

Alzheimer's disease (AD) is a progressive neurodegenerative disorder characterized by cognitive decline and impaired neurocognitive function. It is pathologically defined by neuronal degeneration, loss of neural tissue, and hallmark features such as neurofibrillary tangles (NFT) and amyloid‐beta (Aβ) plaques. Currently, limited treatments exist to halt or slow their progression. Xanthohumol (XH) and Quercetin (Qu) have been reported to exhibit neuroprotective properties against AD by reducing acetylcholinesterase (AChE) activity, mitigating amyloid‐beta accumulation, alleviating oxidative stress, suppressing neuroinflammation, and improving mitochondrial function

**Method:**

The pharmacodynamic studies were conducted using the Morris water maze test to evaluate the cognitive performance of rats and assess the progression of AD. The experimental animals were treated with varying doses of XH and Qu, both individually and in combination. Biochemical assessments were performed to measure key AD markers, including AChE activity, Aβ levels, antioxidants such as catalase, glutathione, and superoxide dismutase and inflammatory mediators such as TNFα, IL‐6 and IL‐8. These assays were conducted to investigate the neuroprotective and anti‐inflammatory properties of the XH and Qu combination. Statistical analysis was applied to compare the outcomes across different groups and determine the most effective treatment regimen.

**Result:**

Pharmacodynamic evaluations of cognitive functions in rats demonstrated notable enhancements in cognition after the administration of XH and Qu in combination. Moreover, biochemical analysis indicated that the combination of XH and Qu significantly reduced AChE activity, Aβ levels, oxidative stress, and neuroinflammation. Among the post‐treated groups, a high dose of XH and Qu was the most effective treatment.

**Conclusion:**

The combination of XH and Qu served as a therapeutic approach to the management of AD. Therefore, our study suggests a potential neuroprotective strategy of XH and Qu with its beneficial effects on AD.

